# “This Pandemic Is Making Me More Anxious about My Welfare and the Welfare of Others:” COVID-19 Stressors and Mental Health

**DOI:** 10.3390/ijerph18115680

**Published:** 2021-05-26

**Authors:** Ramey Moore, Melissa J. Zielinski, Ronald G. Thompson, Don E. Willis, Rachel S. Purvis, Pearl A. McElfish

**Affiliations:** 1 College of Medicine, University of Arkansas for Medical Sciences Northwest, Fayetteville, AR 72703, USA; RameyMoore@uams.edu (R.M.); DEWillis@uams.edu (D.E.W.); RSPurvis@uams.edu (R.S.P.); 2 Department of Psychiatry and Behavioral Sciences, University of Arkansas for Medical Sciences, Little Rock, AR 72205, USA; MJZielinski@uams.edu (M.J.Z.); RGThompson@uams.edu (R.G.T.J.); 3 Department of Psychological Science, University of Arkansas, Fayetteville, AR 72703, USA

**Keywords:** COVID-19, mental health, anxiety and depression, pandemic, stressors, social environment

## Abstract

COVID-19 and subsequent social distancing guidelines have changed many aspects of people’s daily lives including the way that they interact within their social environment. Pandemics are inherently social phenomena, and public health measures intended to curtail transmission of COVID-19 (e.g., quarantine and social distancing) have consequences for individuals with anxiety and depression. Using qualitative methods, respondents with previously diagnosed anxiety or depression identified ways in which COVID-19 affected their symptoms at multiple levels of the social ecological model (SEM). Key themes reported were organized following the SEM. Emergent themes at the individual level are isolation/loneliness, fear of contracting COVID-19, and uncertainty about the future. Themes at the interpersonal level are: fears of family contracting COVID-19, separation from family members, and domestic relationships. Themes at the level of community and societal stressors are: employment, community and societal systems, media, and the COVID-19 pandemic. Our findings demonstrate the ways that mental health, physical health/safety, and social environments are interrelated in the experience of COVID-19 for individuals diagnosed with anxiety or depression. These findings make a significant contribution to the literature as this is the first article to document mental health stressors related to the COVID-19 pandemic among individuals with diagnosed anxiety and depressive disorders.

## 1. Introduction

The World Health Organization officially declared the COVID-19 outbreak a pandemic on 11 March 2020 [1]. The effects of the COVID-19 pandemic extend beyond those on physical health, permeating mental health as well [2,3,4,5]. The concepts of stress and psychological stressors are used to name the effects of the social and physical environment that challenge the capabilities and resources of individuals [6]. The term ‘stressor’ is commonly used to refer to challenges or situations (e.g., any stress stimuli) which have some effect, usually negative, on a person [7].

Recent studies on the prevalence of depression and anxiety symptoms among adults in the United States (US) during COVID-19 have found that generalized anxiety disorder symptoms have significantly risen across all severity levels [8], and the prevalence of depression symptoms has increased three-fold [9]. Individuals with lower social resources, lower economic resources, and greater exposure to increased stressors (e.g., job loss) reported a greater burden of depression symptoms [9]. Evidence from research conducted in Africa [10], Asia [11], Europe [12], the Middle East and North Africa [13], North and South America [4,14], and Oceania [15] documents the multidimensional and interconnected psychological, social, and ecological effects of COVID-19 on mental health, particularly among those with pre-existing anxiety and depressive disorders [16].

Contagious diseases are inherently social phenomena. Social relationships can support physical and mental well-being, and evidence links isolation and low-quality social relationships to an array of mental and physical health conditions [17]. Evidence from other contagious disease epidemics [18] demonstrates that public health measures (e.g., quarantining and social distancing) that reduce the spread of these diseases can have detrimental effects on mental health. There is an emerging qualitative literature on the impacts of COVID-19 on mental health; however, prior literature has only focused on health care workers [19] and special populations such as pregnant women [20], children [21], college students [22], and the elderly [23].

While emerging quantitative research has shown a strong association between the COVID-19 pandemic and anxiety and depression symptoms [16], no prior qualitative studies have explored how the COVID-19 pandemic affects people with pre-existing anxiety and depression symptoms.

### The Current Study

The social ecological model (SEM) is a well-established framework that has been used to analyze the multidimensional interactions among individuals and their social contexts across various levels, ranging from interpersonal to societal systems [24,25]. We apply the SEM as a conceptual framework to examine the impact of COVID-19 stressors on the mental health of adults with pre-existing depressive and anxiety disorders. We focus on these two disorders because they are highly prevalent in the general US population, their prevalence has significantly increased since the COVID-19 pandemic started, they are a leading cause of disability in the US and globally, and they are sensitive to social and ecological determinants of health [26].

The full mental health effects of COVID-19 are not yet well understood, especially for individuals with diagnosed depressive and anxiety disorders. No prior studies have described the experiences of the COVID-19 pandemic on individuals with depressive and anxiety disorders. This study seeks to fill that gap using qualitative methods and a large, diverse sample. Through the lens of the SEM, the purpose of this qualitative study is to describe the lived experiences of individuals with previously diagnosed anxiety and depressive disorders during COVID-19. Understanding these experiences as described in respondents’ own words will aid in developing effective interventions to combat the negative mental health effects experienced during the COVID-19 pandemic by those with anxiety and depression.

## 2. Materials and Methods

### 2.1. Study Design and Sample

The study sample was drawn from participants in the ARresearch research registry, a volunteer research participant registry of individuals who agree to be contacted with opportunities to participate in research and are representative of the ethnic and racial diversity of Arkansas [27]. Participants in ARresearch (*n* = 4431) were sent a recruitment email inviting them to participate in a study about their experiences and perceptions related to the COVID-19 pandemic. Survey responses were collected during July and August of 2020. After invalid and undeliverable email addresses (*n* = 354) were removed, 4077 recruitment emails were sent to potential respondents. Inclusion criteria included respondent verification that they were 18 years of age or older and that respondents resided, were employed, or accessed health care in Arkansas. Screening questions (respondent first and last names, date of birth, and email address) were used to prevent duplicates among potential respondents. A gift card ($20 value) was provided as remuneration for respondents who completed the survey. There were 1288 respondents (31.6% response rate). Among those 1288, 424 respondents indicated they had prior or current diagnoses of anxiety disorders or depression disorders, or a diagnosis of both anxiety and depression disorders. These 424 respondents are included in our analytical sample.

### 2.2. Data Collection

This study used a qualitative descriptive design [28,29]. Qualitative and participant descriptive data were collected through an online instrument. Research Electronic Data Capture (REDCap), a widely used web-based software, was used to capture participant consent and study responses [30,31]. All study materials and procedures were approved by the University of Arkansas for Medical Sciences (UAMS) Institutional Review Board (IRB#261226).

### 2.3. Measures

Selected items from the Behavioral Risk Factor Surveillance System survey [32] assessed respondent demographics (age, sex, race, income). Respondents were also asked whether or not they had ever been: (1) “diagnosed with any depressive disorder (e.g., major depression)” and/or (2) “diagnosed with any anxiety disorder (e.g., panic, generalized anxiety, and post-traumatic stress disorders)”. Respondents then rated their mental health over the two weeks prior to taking the survey on a 5-point Likert scale ranging from *Poor* to *Excellent*. Four open-ended questions prompted respondents to describe their experiences and perceptions related to their mental health during the COVID-19 pandemic. Respondents were asked the following open-ended questions: “Please tell us how COVID-19 has affected your daily life”, “What other source(s) of stress have you experienced during the COVID-19 outbreak?”, “Please tell us how COVID-19 has affected your physical and/or mental health”, and “Is there anything else you would like to tell us about how COVID-19 has affected your physical and/or mental health?”.

### 2.4. Analytic Strategy

Descriptive data documents participant characteristics. A qualitative descriptive approach using the SEM as an analytical framework was used to analyze the responses to open-ended questions. The SEM accounts for different scales of social environments and is well suited to analyzing open-ended questions. Thus, the SEM was used to define the top-level codes derived from the nested scales of analysis defined within the model: a descriptive design synthesizes participants lived experiences and helps capture the meaning that participants attribute to their experiences [33]. Responses were coded with an analytical focus on experiences that respondents described as increasing the symptoms of their anxiety and depression disorders. Quotes are organized within the theme that the quote best represents; however, participants’ comments were multifaceted, and they often discussed multiple, interrelated experiences within the same sentence.

All responses were reviewed by two qualitative researchers who then developed the codebook. Analysis summaries were critically reviewed by a team of five researchers including two clinical psychologists to ensure data, as well as illustrative excerpts, were extracted and categorized within the relevant thematic domain [34]. The research team reviewed the data, codebook, and excerpts to ensure analytic rigor and reliability, following standard qualitative research procedures for codebook development and identification of emergent themes [34,35]. All responses were coded using MAXQDA, a qualitative data analysis tool developed and supported by VERBI software [36]. Divergences in interpreting data were discussed by the research team and resolved using a consensus model. The research team has retained the punctuation and capitalization of the typed responses from respondents to ensure consistency with respondents’ actual words and in their own voice. Representative quotes were collated and statements which best reflect emergent themes were chosen by consensus among the research team [34]. Respondents provided over 1000 responses to open-ended questions; therefore, only the most representative quotes are presented [37].

## 3. Results

### 3.1. Participant Characteristics

Table 1 provides descriptive characteristics of the study sample. Most respondents were between 35 and 64 years of age (66.0%), women (86.3%), and made less than $50,000 per year (57.5%). The majority of the sample reported co-occurring depression and anxiety disorders (65.1%). One-third (37.5%) of respondents reported that their mental health was somewhat poor or poor for the two weeks prior to their response.

### 3.2. Qualitative Results

A priori themes are derived from the SEM: the individual level, the interpersonal level, and a combined community and societal level. Secondary themes emerged within each of the a priori SEM levels.

### 3.3. Individual Stressors

Emergent themes at the individual level are isolation/loneliness, fear of contracting COVID-19, and uncertainty about the future.

#### 3.3.1. Isolation/Loneliness

Isolation and loneliness were reported as increasing anxiety and depression. Respondents stated that they were “more depressed because I am isolated and alone”. Isolation and loneliness were discussed in relationship to both social distancing practices and the concomitant reduction in physical contact from loved ones. Respondents noted the loss of social contact. For example, one stated that the pandemic “affected me emotionally because of the social distancing”, which respondents related to the intensification of “depression symptoms”. Respondents discussed their concerns about the lack of physical closeness and stated, “although social distancing has become a regular practice along with taking safety measures in public, it is still emotionally trying still not being able to obtain physical touch/comfort”. Respondents noted social “meetings are held by video, which is depressing” which indicates that videoconferencing may not provide the needed social connection or alleviate feelings of isolation. 

#### 3.3.2. Fear of Contracting COVID-19

Respondents reported anticipatory anxiety related to COVID-19, with the disease causing “increased anxiety” about contracting COVID-19. Respondents stated that the COVID-19 pandemic “has increased my anxiety because I’m worried about getting sick”. One respondent described “I’m anxious that we are going to be sick”. Another respondent stated her “mental health has been affected because of anxiety of getting sick”. Other respondents noted “anxiety is worse” because they feared “getting COVID-19”. Other respondents described the pandemic worsening behaviors associated with their mental illness. For example, a respondent who identified as having obsessive-compulsive disorder described the “time spent preparing when I have to go out” including “washing clothes and showering when I come home” and “disinfecting anything that comes into my home” was “basically my worst nightmare coming true” to which this respondent attributed increased symptoms of her obsessive-compulsive disorder.

#### 3.3.3. Uncertainty about the Future

Respondents identified greater uncertainty about the future (stemming from the COVID-19 pandemic) as a mental health stressor at the individual level. Respondents stated that they “feel much more anxiety worrying about the unknown”, and they feel “increased anxiety from so much unknown”. Respondents also noted “the uncertainty about the future kinda gets me more depressed”. Respondents discussed how thinking about the future affected anxiety and depression symptoms. As one respondent noted, they felt “super depressed and anxious lonely and a bit hopeless” because “there’s nothing to look forward to”. Other respondents noted a “concern for our future” directly linked to experiences of “mental distress” and “depression”. Some respondents identified “a lot of anxiety about no knowing the long-term effects of the disease”.

### 3.4. Interpersonal Stressors

At the interpersonal level, the primary themes are: fears of family contracting COVID-19, separation from family members, and domestic relationships.

#### 3.4.1. Fears of Family Contracting COVID-19

Fear of family members contracting COVID-19, either in general or due to transmission from participants directly, was identified as a stressor at the interpersonal level. Respondents stated “anxiety is always high trying to protect myself and my family members who are at a higher risk”. One respondent stated “fear of spreading [COVID-19] to my grandfather” is “a large contributing factor in my depression”. Other respondents noted “fear for the life of my family” as being among the stressors for their anxiety and depression disorders, and “my husband is in very poor health and the concern for him has been one of my biggest stressors”. Respondents often noted that their anxiety about COVID-19 centered not on themselves. One respondent stated “I feel very anxious due to worry about my family more so than myself”.

#### 3.4.2. Separation from Family Members

Separation from family was identified as a stressor of mental health at the interpersonal level. Respondents described how “decreased interaction with family” negatively affects anxiety and depression symptoms. Separation from family was also noted by a respondent who stated “I feel like I maybe getting depressed from staying home and not being able to see family”. In addition to those who reported an increase in anxiety and depression symptoms due to a lack of contact and support from family members, respondents also described detrimental mental effects coupled with guilt from not being able to help family members. One respondent described their experience as “stress, can’t sleep, anxiety, depression, crying, worrying, feeling of guilt because can’t help my family”. One respondent stated a mental health crisis was intensified due to being “unable to physically reach out for help from family and friends”. Another respondent reported anxiety due to “less/no contact with most family”.

#### 3.4.3. Domestic Relationships

Domestic relationships were also identified as a mental health stressor during COVID-19. One respondent described the effect of the pandemic on her mental health as “responsible for my own and my boyfriend’s relapse” which “ruined a lot of what was good in my relationship”. Another respondent indicated an increased dosage of her anti-depressant medication resulted in negative effects to her “sex life with my husband”. Other respondents noted difficulties in their intimate relationships. A respondent stated “do to being a higher risk individual, I was sent home to work only to lose my home due to marital strife” with the eventual result being an in-patient hospital stay for psychiatric care. Increased violence in the home was also identified as a stressor. One respondent identified herself as a “victim of recent domestic abuse”, which she described as “mentally extremely difficult to deal with having to be with your abuser”. Even for respondents who are not in abusive households, communication between partners was also identified as a stressor affecting anxiety and depression. One respondent reported her mental health as being “devastated”, stating she “scared [her] psychiatrist on a telemed visit” due to fatigue and severe weight loss. This respondent also described “spiraling into depression” because of problems with her boyfriend.

### 3.5. Community and Societal Stressors

Emergent themes at the level of community and societal stressors are: employment, community and societal systems, media, and the COVID-19 pandemic in general.

#### 3.5.1. Employment

Respondents identified employment-related stressors as affecting their anxiety and depression symptoms. Several respondents reported fear of unintentionally bringing COVID-19 back home to their families because of work as essential employees. One specific interpersonal stressor is related to the theme of fears of family contracting COVID-19 and is specifically linked to their employment. A respondent who identified herself as a hair dresser stated her “anxiety is always high trying to protect myself and my family members who are at a higher risk”. Others reported “I have kids and family with weaken immune systems and still have to work (healthcare) and that itself affect my mental status a lot”, as well as worry “I may possibly bring something back to my family”. Respondents stated feeling stress “because so much to do at work and worry about bringing it home to my mom, or getting it myself”, linking both fears of contracting COVID-19 at work with anxiety related to her work. One respondent who works as an attorney said that “courts across the state have reopened” and “I’m forced to go to these courts where limited precautions are taken”, which makes him “very nervous and anxious”.

Beyond protecting themselves, respondents reported increased work demands and changes in employment because of COVID-19 and reported how this affected their anxiety and depression symptoms. A respondent who identified herself as working in an academic health care facility noted significant increase in work hours, and feeling “overworked” and “overtired” related to “more stress and anxiety due to new work responsibilities”. A respondent who works in an emergency room setting linked feelings of exhaustion to her “history of PTSD”, for which she changed her psychiatric medications to “decrease night terrors and help depression”.

Respondents noted effects on anxiety and depression symptoms related to changing and increasing job duties, leaving jobs, or losing hours at a current job. One respondent noted “I had to change where I worked due to loss of hours thus taking a $14/h pay cut”. This respondent linked “major financial losses” with intensified symptoms of “depression”. Respondents also reported the effect of employment stressors on other members of their household. One respondent stated her “husband committed suicide due to the stress of closing his dental business related to COVID”.

#### 3.5.2. Community and Societal Systems

Respondents reported the effects of the COVID-19 pandemic on community and societal systems as a stressor directly affecting symptoms of anxiety and depression. Respondents described worrying about their “community” at large and anxiety concerning “what this [pandemic] will do to our country”. Respondents also noted specific mental health effects, such as increased anxiety “as I see rising numbers of Covid-19” coupled with shortages of “supplies such as masks, as well as seeing certain items in the stores disappear from shelves”. Other respondents stated that they were “depressed” due to the community members’ response or lack of response to the pandemic, including specifically “people who think that masks are a conspiracy”. Government responses to the pandemic were noted at the community and societal level as anxiety and depression stressors. Respondents specifically linked “mental distress” and “depression” to “distrust of government entities” and the way that government was handling the COVID-19 pandemic. One respondent reported that the COVID-19 pandemic “has caused mental distress, anger, distrust of government entities, depression”.

#### 3.5.3. Media

Respondents identified engagement with media, including traditional and social media, regarding COVID-19 as a stressor affecting their anxiety and depression symptoms. Respondents stated that they feel “more anxious” due to “reading so much of the news on Facebook and Yahoo”. A respondent reported “my anxiety is terrible and I feel trapped”, because “I spend many hours reading news online and ruminating”. One respondent noted “I had to go in-patient for my depression and anxiety due to the stress of COVID-19 news”. Another respondent noted a relapse of alcoholism which she attributed partially to “worrying from watching too much news and social media”. Respondents also described stress and anxiety from not knowing which news “is correct about” COVID-19 and whether or not they could trust news stories as “factual”.

#### 3.5.4. COVID-19 Pandemic

Respondents discussed the COVID-19 pandemic broadly as a source of stress affecting anxiety and depression symptoms. Respondents stated that they “suffered from anxiety and depression about the pandemic”. Several respondents related an increase in symptoms related directly to the pandemic and stated their “anxiety and depression are much worse than normal due to the added stress of the pandemic”. This intensification of symptoms attributed to the pandemic was echoed by a respondent who stated “my general anxiety disorder went into high gear because of the pandemic”. Another respondent stated “anxiety and depression are much worse than normal due to the added stress of the pandemic”. Another respondent stated “I suffer from depression and the virus has made it worse”.

## 4. Discussion

COVID-19 and subsequent social distancing guidelines have changed many aspects of people’s daily lives including the way that they interact within their social environment. Using qualitative methods, respondents with previously diagnosed anxiety or depression identified ways in which COVID-19 affected anxiety and depression symptoms at multiple levels of the SEM. Respondents described COVID-19 stressors in their own words. Key themes that emerged at the individual level were isolation and loneliness, fear of contracting COVID-19, and uncertainty about the future. These findings are consistent with the literature regarding the negative effects of social and physical separation from family members, as part of COVID-19 social distancing guidelines among the general population and for special populations (e.g., the elderly or young people) [8,38,39,40].

Fear of contracting COVID-19 was described as exacerbating symptoms of anxiety and depression. These findings are consistent with literature reporting rising fears related to the COVID-19 pandemic, including the fear of contracting COVID-19 [4,41], as well as literature describing increases in anxiety related to health during the COVID-19 pandemic for those without diagnosed mental illness [42]. This is the first study to document these fears among those with diagnosed depression and anxiety disorders.

This study’s finding regarding the fear of contracting COVID-19 coupled with uncertainty about the future are consistent with Ulrich Beck’s risk society model, and this study presents the first confirmation that the pandemic’s effects are felt by individuals as part of the inherent vulnerabilities of risk society [43,44].

At the level of interpersonal relations, key themes included family contracting COVID-19, separation from family members, and domestic relationships. Fears of family contracting COVID-19 are consistent with emerging literature on the subject [45]. The specific stress of social distancing reported by respondents is also consistent with findings in the literature [46]. The findings regarding the added stress of domestic relationships is consistent with prior literature that links increases in domestic stress and violence during natural disasters [47] and the COVID-19 pandemic [48]. The wide range of domestic relationship stressors demonstrates the need for additional family and social support during pandemics especially for those with mental health concerns. Expansion of telehealth options for mental health, coupled with informational campaigns pointing individuals to existing telehealth resources, could represent one means for addressing family and social support needs. Integrating domestic and intimate partner violence safeguards into public health measures, disaster response efforts, and disaster recovery measures also makes up an important part of holistic public health measures to address these issues [48].

At the community and societal levels, key themes were employment, community and societal systems, media, and the COVID-19 pandemic in general. The findings related to the emergent theme of employment are consistent with literature on the effects of job stress on mental health [49,50,51,52]. The connection between job stress about becoming infected with COVID-19 and anxiety about infecting household members provides new understandings about the ways that job stress affects mental health during the COVID-19 pandemic. Respondents discussed the effects of media, both traditional and social, on symptoms of anxiety and depression. This is consistent with studies that highlight how exposure to disaster-related news media as well as higher levels of social media use are associated with negative effects on mental health [53,54,55]. This is the first study to document the negative mental health effects of media during COVID-19 among those diagnosed with anxiety and depression. Respondents also noted that COVID-19, and the pandemic as a whole, directly affected their symptoms of anxiety and depression. This is consistent with emerging literature on the effects of the pandemic on individuals with mental illness, where the COVID-19 pandemic is reported as affecting mental health in myriad ways [56]. There are some indications that economic relief programs serve to mitigate or prevent trauma from the economic and social effects of the pandemic and the exacerbating effects of public health responses on symptoms of anxiety and depression [57].

### 4.1. Limitations

This study does have limitations that should be considered. The sample was drawn from a research registry in the state of Arkansas, which allows for a large and diverse sample; however, this sample may not be generalizable to the population as a whole. Additionally, respondents were not asked to rate their current mental health state compared to their mental health prior to the pandemic, which potentially limits more nuanced conclusions from the data. Diagnoses were self-reported, and a diagnosis was not confirmed in respondents’ medical records. The use of written electronic data capture allowed respondents to reply with a greater degree of confidentiality than with individual interviews or group interviews but also prevented clarifying questions and probes.

### 4.2. Theoretical and Practical Implications

There are several implications for the current study, including both theoretical and practical issues. The SEM represents a powerful tool for incorporating a contextual exploration of health; however, the SEM has not been fully leveraged in studies of depression and anxiety disorders. Applications of the SEM opens up new modalities for understanding and addressing mental health at multiple social-ecological levels. This focus on the social ecology of mental health invites a focus on individuals within their social, cultural, and political contexts. Practically, the SEM can be used to generate more holistic health promotion and to shape interventions that operate at multiple levels, not merely at the level of individuals diagnosed with anxiety and/or depression. There are also implications for future public health measures related to epidemics and pandemics. This research suggests that future public health responses need to account for the potential mental health effects of quarantine and social distancing. This research points to potential levels for interventions and also to the need for more research concerning practical interventions for mental health during pandemic conditions.

### 4.3. Future Research Directions

Several areas of further research are indicated based on the findings of this study. First, research exploring the role of mental health stressors among persons without a diagnosis of depression and anxiety, and comparing those findings with the current study, would provide a broad view of mental health during the pandemic. In addition, comparisons across gender differences would provide a more textured picture for how stressors may work differentially across intersectional identities. Finally, this research suggests that socio-economic status may affect experiences of mental health stressors, which would require further study to explore.

## 5. Conclusions

This article makes a significant contribution to the literature as the first article to document stressors related to the COVID-19 pandemic among people with pre-existing anxiety and depressive disorders. This study is novel in its application of qualitative methods to explore mental health stressors in a large, diverse sample. The insight into lived experiences provides a basis for mental health interventions to mitigate a COVID-19-related mental health crisis. Efforts to mitigate a ‘second pandemic’ related to mental health must be implemented. This is complicated by the fact that the most effective interventions for pandemics are disruptive to the systems that support mental health and mental hygiene, including support from social connections and other coping mechanisms [8,17,58].

Findings from this study underscore the need to expand mental health services to include the launching of emotional support and treatment referral hotlines to connect uninsured and underinsured individuals with anxiety and depression to affordable care. Policies requiring health insurers to waive out-of-pocket costs for in-network mental health care and relaxing regulations for the provision of mental health services via telemedicine should be implemented to alleviate the impact of business closures and social distancing on access to needed care. Overall, our findings demonstrate the ways that mental health, physical health and safety, and social environments are interrelated in the experience of COVID-19. Interventions focused on mental health must account for the isolating effects of quarantine and stay-at-home orders.

## Figures and Tables

**Table 1:** Descriptive statistics for study sample.

Demographics	Total	Percentage (%)
Age (*n* = 424)		
18–34	111	26.2
35–64	280	66.0
65 plus	33	7.8
Sex (*n* = 422)		
Male	58	13.7
Female	364	86.3
Race/Ethnicity (*n* = 424)		
Non-Hispanic Black	37	8.7
Non-Hispanic White	343	80.9
Non-Hispanic Other	15	3.5
Hispanic/Latino	29	6.8
Income (*n* = 398)		
<$25,000	125	31.4
$25,000 to <$50,000	104	26.1
$50,000 to <$75,000	84	21.1
$75,000 plus	85	21.4
Anxiety (*n* = 424)		
Yes	354	83.5
No	67	15.8
Don’t know/Not sure	3	0.7
Prefer not to answer	0	0.0
Depression (*n* = 424)		
Yes	312	73.6
No	108	25.5
Don’t know/Not sure	3	0.7
Prefer not to answer	1	0.2
Both depression and anxiety (*n* = 424)	276	65.1
Mental Health (*n* = 424)		
Excellent	21	5.0
Somewhat good	102	24.1
Average	139	32.8
Somewhat poor	114	26.9
Poor	45	10.6
Don’t know/Not sure	3	0.7
